# Increased Matrix Metalloproteinase (MMPs) Levels Do Not Predict Disease Severity or Progression in Emphysema

**DOI:** 10.1371/journal.pone.0056352

**Published:** 2013-02-18

**Authors:** Jeanine M. D’Armiento, Monica P. Goldklang, Andrew A. Hardigan, Patrick Geraghty, Michael D. Roth, John E. Connett, Robert A. Wise, Frank C. Sciurba, Steven M. Scharf, Jincy Thankachen, Monirul Islam, Andrew J. Ghio, Robert F. Foronjy

**Affiliations:** 1 Division of Pulmonary, Allergy and Critical Care Medicine, Columbia University Medical Center, New York, New York, United States of America; 2 Division of Pulmonary and Critical Care Medicine, St. Luke’s-Roosevelt Health Sciences Center, New York, New York, United States of America; 3 Division of Pulmonary and Critical Care Medicine, University of California Los Angeles, Los Angeles, California, United States of America; 4 Department of Biostatistics/CCBR, University of Minnesota, Twin Cities, Minnesota, United States of America; 5 Division of Pulmonary and Critical Care Medicine, Johns Hopkins University, Baltimore, Maryland, United States of America; 6 Division of Pulmonary, Allergy, and Critical Care Medicine, University of Pittsburgh, Pittsburgh, Pennsylvania, United States of America; 7 Division of Pulmonary and Critical Care Medicine, University of Maryland, Baltimore, Maryland, United States of America; 8 National Health and Environmental Effects Research Laboratory, Environmental Protection Agency, Research Triangle Park, North Carolina, United States of America; Boston University Medical Center, United States of America

## Abstract

**Rationale:**

Though matrix metalloproteinases (MMPs) are critical in the pathogenesis of COPD, their utility as a disease biomarker remains uncertain. This study aimed to determine whether bronchoalveolar lavage (BALF) or plasma MMP measurements correlated with disease severity or functional decline in emphysema.

**Methods:**

Enzyme-linked immunosorbent assay and luminex assays measured MMP-1, -9, -12 and tissue inhibitor of matrix metalloproteinase-1 in the BALF and plasma of non-smokers, smokers with normal lung function and moderate-to-severe emphysema subjects. In the cohort of 101 emphysema subjects correlative analyses were done to determine if MMP or TIMP-1 levels were associated with key disease parameters or change in lung function over an 18-month time period.

**Main Results:**

Compared to non-smoking controls, MMP and TIMP-1 BALF levels were significantly elevated in the emphysema cohort. Though MMP-1 was elevated in both the normal smoker and emphysema groups, collagenase activity was only increased in the emphysema subjects. In contrast to BALF, plasma MMP-9 and TIMP-1 levels were actually decreased in the emphysema cohort compared to the control groups. Both in the BALF and plasma, MMP and TIMP-1 measurements in the emphysema subjects did not correlate with important disease parameters and were not predictive of subsequent functional decline.

**Conclusions:**

MMPs are altered in the BALF and plasma of emphysema; however, the changes in MMPs correlate poorly with parameters of disease intensity or progression. Though MMPs are pivotal in the pathogenesis of COPD, these findings suggest that measuring MMPs will have limited utility as a prognostic marker in this disease.

## Introduction

In COPD, MMPs contribute to airway obstruction by triggering dysfunctional matrix remodelling [1] and inducing inflammation [2]. Tissue destruction in this disease results from an imbalance in protease/antiprotease activity such as MMPs and their inhibitors, tissue inhibitors of metalloproteinases (TIMPs) [3]–[5]. The collagenase MMP-1, the gelatinase MMP-9 and the metalloelastase MMP-12 are of particular interest in emphysema pathogenesis. MMP-1 is up regulated in alveolar walls and macrophages in human emphysema [6], [7] and the transgenic expression of human MMP-1 in mice, which lack the gene for MMP-1, generates adult-onset emphysema, associated with a loss of lung compliance [8]. Similarly, MMP-9 levels are elevated in the lung [5] and alveolar macrophages of COPD patients [4], [9]. Indeed, circulating monocytes of advanced emphysema subjects have the highest levels of MMP-9 production [10] and the expression of this protease in alveolar macrophages causes progressive adult-onset emphysema in mice [11]. The metalloelastase, MMP-12, was shown to be required for cigarette smoke-induced emphysema in mice [12]. Furthermore, sputum MMP-12 concentrations correlate with the extent of emphysema as determined by CT [13] and polymorphisms in the MMP-12 promoter have been associated with changes in lung function [14], [15]. Given their importance, this study aimed to determine whether MMP-1, -9, -12 and TIMP-1 (tissue inhibitor of matrix metalloproteinase-1) levels measured in the bronchoalveolar lavage fluid (BALF) and plasma of advanced emphysema subjects were predictive of the severity and future course of the disease.

## Methods

### Selection Criteria for Study Participants

Emphysema BALF samples were obtained from baseline measurements of Feasibility of Retinoids for the Treatment of Emphysema (FORTE) trial participants [16]. Briefly, subjects were over 45 years of age with an FEV_1_ 25 to 80% of predicted, a reduced diffusing capacity of the lung for carbon monoxide (DLco) and emphysema documented on chest CT scan. Of note, subjects were clinically stable and were excluded if there was history of recent history of exacerbation of systemic corticosteroid use. All included emphysema participants had abstained from tobacco use for at least 6 months, confirmed by a serum cotinine level of less than 20 ng/ml. Analysis included 101 FORTE trial participants with moderate to severe emphysema, 17 current smokers with normal pulmonary function as well as 72 volunteers with no significant respiratory disease and no smoking history. Written consent was obtained from all study participants and the trial was approved by the institutional review boards of Columbia University Medical Center, the University of Pittsburgh, the University of California at Los Angeles Medical Center, the University of California at San Diego Medical Center, Boston University Medical Center and Long Island Jewish Medical Center.

### Outcomes Measurements

Pulmonary function tests were conducted by qualified personnel in accordance with American Thoracic Society standards [17], with predicted values according to Hankinson et al [18] and Crapo et al [19]. The overall respiratory status was assessed with the St. George Respiratory Questionnaire (SGRQ) [20]. Radiology technologists were trained in a standard imaging protocol across the various study centers as previously reported [16]. A water phantom (GE; Milwaukee, WI) was scanned at each center, and the reconstruction kernels were selected to provide equivalent images across institutions [21]. Ten-millimeter collimation was used (no overlap), and images were transmitted in digital format to the Radiology Core. Image analysis was performed to determine lung volume and the percentage of emphysema by density mask (percentage of total lung voxels<− 910 Hounsfield units [HU]) [22]. Density mask scores were not adjusted for CT-measured lung volume due to possible independent effects of treatment on these parameters.

### Bronchoscopy Procedure

Fiberoptic bronchoscopy was performed on an outpatient basis in the endoscopy units of the participating study centers as per standard protocol. Bronchoscopy details for this study have been previously described [16], [23].

### Measurement of Protease Activity

Collagenase and elastase activity was measured from the BALF of non-smoking controls, smokers and emphysema subjects. Collagenase units were calculated as the number of µmoles of leucine liberated per mg of enzyme, by a colorimetric ninhydrin method [24], [25]. Elastase activity was measured by the hydrolysis of N-succinyl-L-Ala-L-Ala-L-Ala-p-nitroanilide (Suc Ala_3_NA) per minute at 25°C and pH 8.0 under the specified conditions [26], [27].

### Processing of BALF and Plasma Samples

BALF and plasma was processed as previously reported [16]. Once obtained the BALF fluid was filtered with a sterile Falcon 100-micron nylon mesh to remove mucus and debris. The fluid was then centrifuged at 200×*g* for 15 minutes at 4°C. The BALF supernatant was aliquoted and immediately stored on-site in a −80°C freezer. Aliquots were shipped frozen from the testing sites to Columbia University for determination of matrix metalloproteinase levels. Approximately 30 ml of blood was obtained via venipuncture into three 10 ml heparinized tubes. These tubes were then centrifuged at 200×*g* for 8 minutes at 4°C. The plasma was transferred into labeled 1.5 ml tubes and stored at −70°C to −80°C on-site until they were ready to be shipped as described above.

### Determination of Matrix Metalloproteinase-1, -9, -12 and Tissue Inhibitor of Metalloproteinase-1 Levels

ELISAs for MMP-1, -9 and TIMP-1 (Amersham Biosciences, Piscataway, NJ, USA) were conducted on BALF and plasma of the entire emphysema cohort (101 subjects). MMP-12 levels were measured in the BALF (N = 66 emphysema subjects) and plasma (N = 49 emphysema subjects) using a Luminex assay (Invitrogen, Carlsbad, CA, USA). Since the original plasma determinations had been only conducted on emphysema subjects, repeat plasma analyses for MMP-1, -9, -12 and TIMP-1 were conducted using Luminex multiplex (Invitrogen, Carlsbad, CA, USA) to compare MMP plasma levels between study emphysema subjects and controls (5 age-matched smokers, 15 age-matched non-smokers and 49 emphysema subjects).

### Statistical Analysis

All data analysis was performed using GraphPad Prism software (La Jolla, CA, USA). Significant differences in levels between the cohorts were assessed by the Kruskal-Wallis one-way analysis of variance by ranks followed by the Dunn’s multiple comparison test. Wilcox signed rank tests were conducted to examine difference between individual cohorts. Mann-Whitney tests were utilized to determine significant differences between quartiles of MMP expression. These non-parametric analyses were chosen due to the skewed distribution of the results. A p-value of <0.05 considered statistically significant. Coefficient of determination and linear regression analyses were conducted to examine the association between MMPs levels as determined by ELISA or Luminex (MMP-12) and key disease parameters.

## Results

### Baseline Patient Characteristics

For the lavage studies, subjects with emphysema were older than the non-smoking controls and smokers without emphysema (Table 1). Plasma samples were well matched by age across the three cohorts. The 101 emphysema subjects exhibited at least moderately severe airway obstruction in pulmonary function testing and had visual evidence of emphysema occupying ≥10% of the lung on CT (Table 2).

**Table 1:** Patient Demographics.

	Non-smokers	Smokers	Emphysema
Number	72	17	101
Age (years)	24.2±3.7	26.1±4.8	65.5±7.5
Gender (Male/Female)	60%/40%	76%/24%	57%/43%
Race (Caucasian/African-American)	88%/12%	76%/24%	100%/0%
Mean Smoking History (years)	0±0	7.8±1.6	58.4±29.9

Data are presented as mean ± standard deviation.

**Table 2:** Emphysema characteristics.

Current smokers	0
Average cigarettes per day prior to smoking cessation	31.1±15
Pack years	58.4±29.9
FEV1	46±12.7
FVC	82.5±14.3
FEV1/FVC	42.2±9.5
Bronchodilator responsiveness	13.1±10.2
Total lung capacity	116.6±15.1
Residual volume	166.6±41.2
DLCO	39.2±11.8
CT % Emphysema (HU<−910)	37.9±12.8

All data are presented as mean ± standard deviation. Data for forced expiratory volume in one second (FEV_1_), forced vital capacity (FVC) total lung capacity, residual volume and diffusion capacity for carbon monoxide (DLCO) are all presented as percent predicted. FEV1/FVC ratio is presented as %. Bronchodilator responsiveness is presented as % change in FEV1.

### MMP Levels in Emphysema, Smokers and Normal Controls

**Figure 1:**
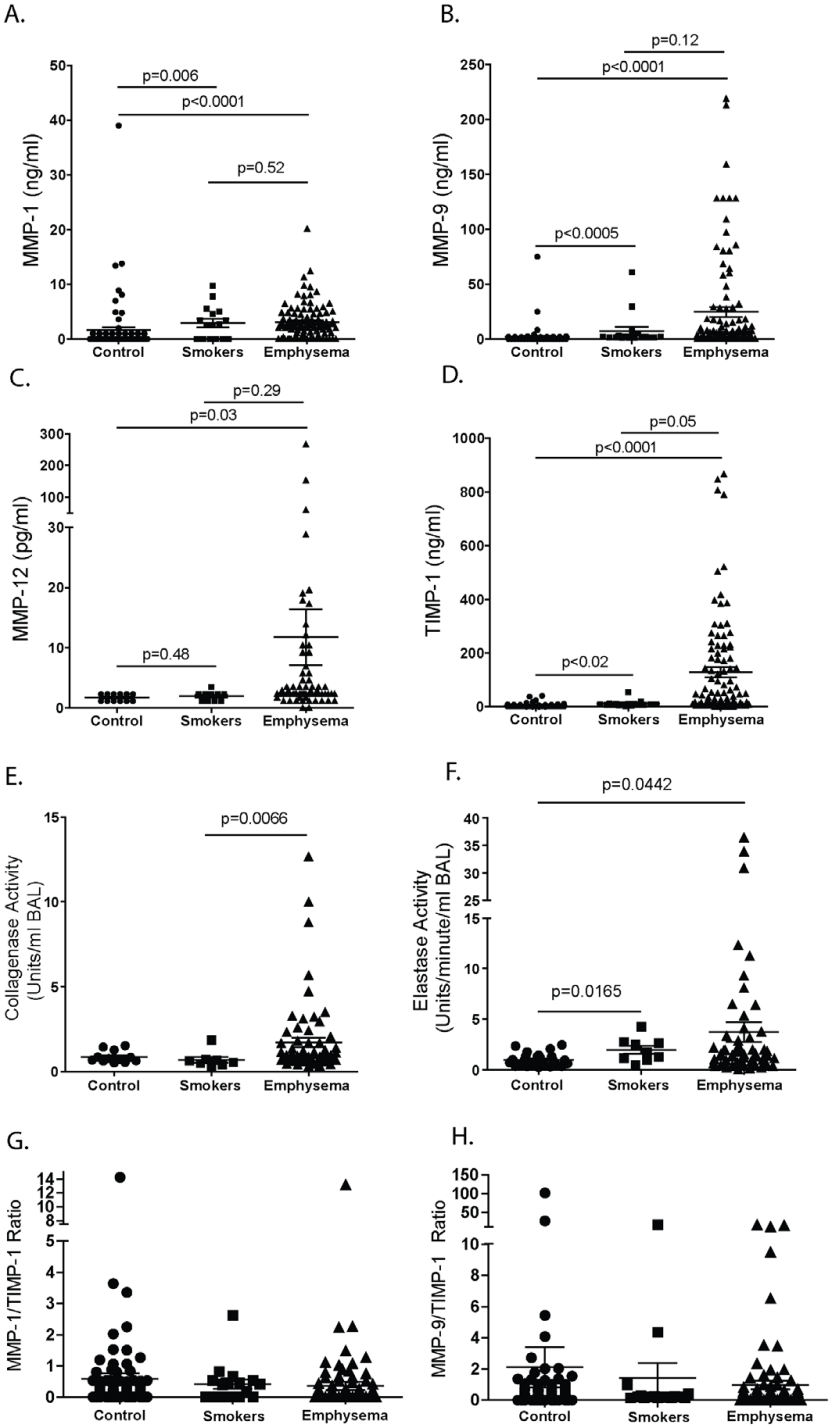
BALF MMP and TIMP-1 protein levels and protease activity in controls, smokers and emphysema subjects. BALF was performed on 72 normal control subjects, 16 smokers, and 101 subjects with moderate to severe emphysema who had refrained from smoking for at least six months. (A) MMP-1, (B) MMP-9, (C) MMP-12 and (D) TIMP-1 protein levels were recorded. p<0.0001 by Kruskal-Wallis one-way analysis of variance by ranks followed by a Dunn’s multiple comparison test. *p* values shown, comparing both treatments connected by a line. (E) Collagenase and (F) elastase activity were measured in the BALF of 26 normal controls, 9 smokers without emphysema and 60 emphysema subjects. Differences between cohorts was assessed by ANOVA; p<0.05. (G) MMP-1/TIMP-1 BALF ratios and (H) MMP-9/TIMP-1 BALF ratios were determined in the BALF of 82 normal controls, 18 smokers without emphysema and 98 emphysema subjects. Differences between cohorts was assessed by ANOVA; p<0.05.

**Figure 2:**
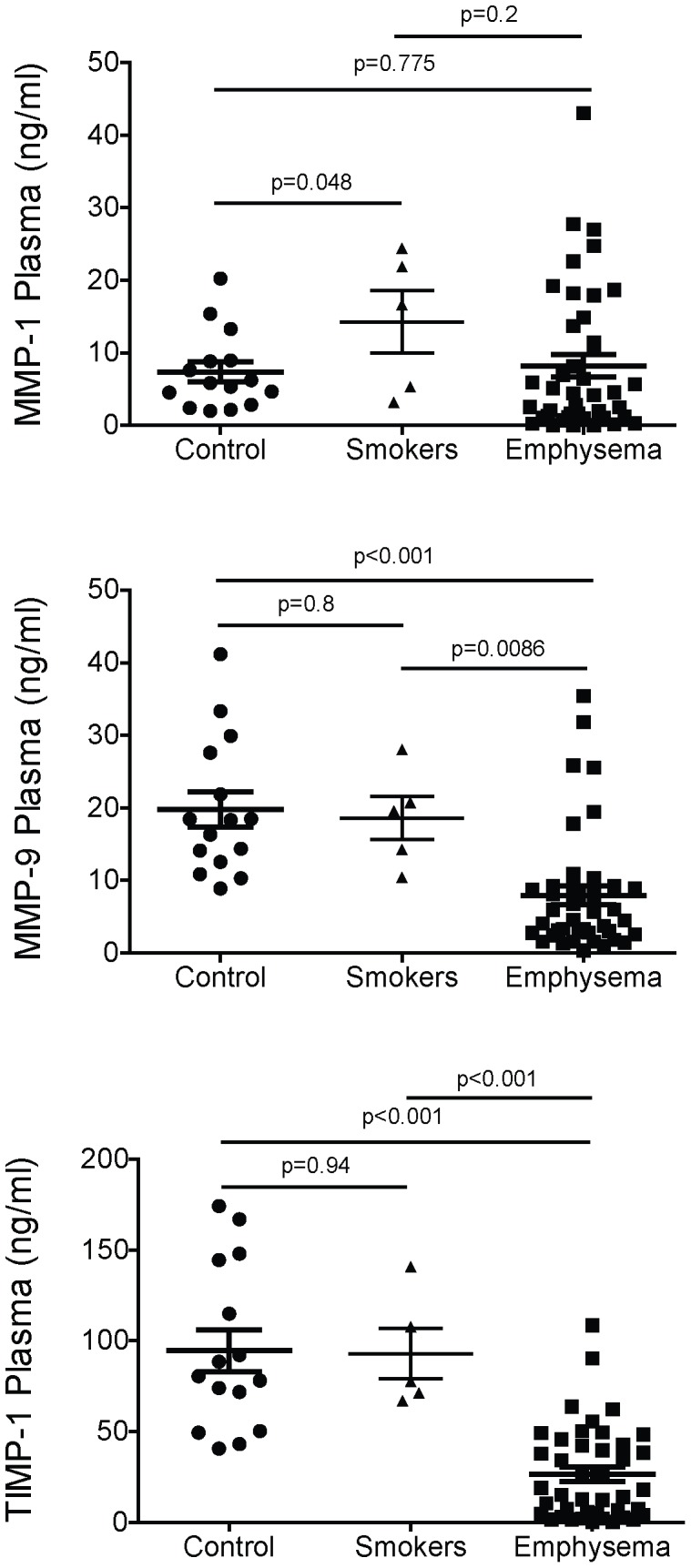
MMP and TIMP-1 levels in the plasma of normal smokers and emphysema subjects. Luminex multiplex assays for MMP-1 (top panel), MMP-9 (center panel) and TIMP-1 (bottom panel) were done on age-matched plasma samples from non-smoking controls, normal smokers and subjects with moderate to severe emphysema. MMP-12 was measured but levels were below the limit of detection.

Significant increases in BALF MMP-1 protein levels (0.8±4.5 ng/ml in control, 3.0±3.0 ng/ml in smokers, 2.7±3.0 ng/ml in emphysema; p<0.0001 by Kruskal-Wallis test, all data are presented as mean ± standard error) (Figure 1A) and MMP-9 protein levels (2.1±9.2 ng/ml in control, 7.5±15.4 ng/ml in smokers, 24.9±44.7 in emphysema; p<0.0001 by Kruskal-Wallis test) (Figure 1B) were detected amongst the three cohorts. Analyses between paired groups showed that MMP-1 and -9 levels in smokers and emphysema subjects were significantly increased compared to non-smoking controls. Though BALF MMP-12 levels were altered amongst the study cohorts (1.7±0.1 ng/ml in control, 2.0±0.3 ng/ml in smokers, 11.8±4.7 ng/ml in emphysema; p<0.004 by Kruskal-Wallis test) (Figure 1C), only controls and emphysema subjects showed a significant intergroup difference. BALF TIMP-1 levels were also altered amongst the three study cohorts (TIMP-1 4.2±7.2 ng/ml in control, 11.5±11.8 ng/ml in smokers, 127.8±189.7 in emphysema; p<0.0001 by Kruskal-Wallis test) (Figure 1D). BALF TIMP-1 levels were higher in smokers and emphysema subjects compared to controls and those with emphysema had even higher levels than active smokers (Figure 1D). Though MMP-1 levels were comparable in smokers and emphysema subjects, only those with emphysema had significantly increased collagenase activity (Figure 1E). Moreover, this increase in activity occurred despite the fact that TIMP-1 levels were notably higher in the emphysema cohort compared to the smoking group. Elastase activity, on the other hand, was increased in both the smokers and the emphysema subjects (Figure 1F). The MMP-1/TIMP-1 (Figure 1G) and MMP-9/TIMP-1 (Figure 1H) ratios were comparable amongst the three cohorts. In comparison to BALF, MMP-1 plasma levels were higher in active smokers than they were in the control or emphysema cohort (Figure 2, top panel) (7.35 ng/ml in control, 14.27 ng/ml in smokers and 8.20 ng/ml in emphysema; p<0.0001 by Kruskal Wallis test) and MMP-9 (19.76 ng/ml in control, 18.6 ng/ml in smokers and 7.93 ng/ml in emphysema; p<0.0001 by Kruskal Wallis test) and TIMP-1 levels (94.49 ng/ml in control, 92.93 ng/ml in smokers and 26.36 ng/ml in emphysema; p<0.0001 by Kruskal Wallis) were significantly lower in the emphysema cohort compared to both the control and smoker groups (Figure 2, center and bottom panels).

### MMP Levels and Disease Parameters in Emphysema

**Figure 3:**
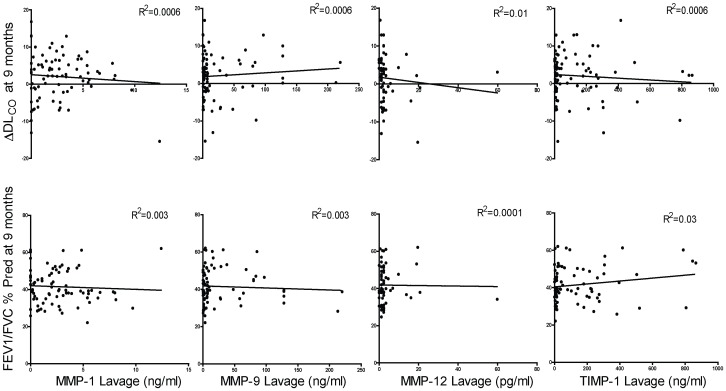
Correlation between MMPs and DL_CO_ or FEV1/FVC % predicted. Linear regression analyses correlated baseline BALF MMP-1, -9, -12 and TIMP-1 lavage levels with change in DL_CO_ or the FEV1/FVC % predicted ratio at 9-month follow up. R^2^ = coefficient of correlation.

**Figure 4:**
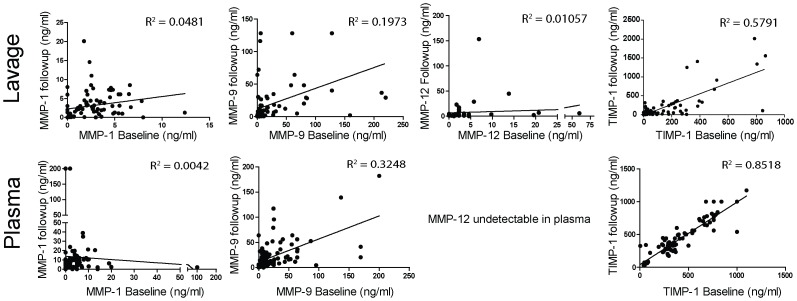
Correlation between baseline and follow up BALF and plasma MMP/TIMP levels. Baseline BALF and plasma protein levels for MMP-1, -9 and TIMP-1 were correlated with follow up MMP/TIMP levels that were collected and measured 3 or 6 months following the baseline tests. The coefficient of determination (R^2^) was calculated for each value at each time point.

To assess correlation with disease severity, BALF and plasma MMP-1, -9, -12 and TIMP-1 levels were broken down into quartiles of protein expression in the emphysema subjects (Table 3). Analyses were then conducted to determine if increasing quartiles of protein expression were associated with age, pack years, FEV1% predicted, FEV1/FVC % predicted, TLC % predicted, CT score or St. George questionnaire total score (Tables 4 and 5). Age was associated with higher quartiles of MMP-9, -12 and TIMP-1 BALF protein levels and the highest quartile of MMP-9 plasma protein expression was associated with increased TLC % predicted. In contrast to a prior study [13], higher MMP-12 levels were associated with lower emphysema scores on CT. However, after adjustment for multiple comparisons, these differences were no longer statistically significant. Linear regression analyses were conducted comparing baseline BALF MMP values with Δ DLCO and FEV1/FVC % predicted at 9 months (Figure 3). No significant associations were found. Similarly, we found no correlation between BALF elastase and collagenase activity and COPD disease parameters (Table 6). Baseline BALF and plasma MMP-1, -9 and -12 levels correlated poorly with follow up measurements performed 3 to 6 months later (Figure 4) despite the fact that the emphysema subjects were in a stable disease state [23]. TIMP-1 levels, on the other hand, demonstrated greater consistency over time with TIMP-1 follow up plasma levels showing a very strong correlation with baseline values.

**Table 3:** Quartiles of MMP protein expression.

	Lavage MMP-1	Lavage MMP-9	Lavage MMP-12	Lavage TIMP-1	Plasma MMP-1	Plasma MMP-9	Plasma TIMP-1
Minimum	0	0	0	0	0	0	39
25^th^ %	1	1	2	12	2	6	238
Median	2	6	2	45	5	13	303
75^th^ %	4	28	6	214	10	29	495
Maximum	12	220	267	865	200	200	1098
Mean	3	27	12	135	9	28	349

Minimum to 25% represented quartile 1 (Q1), 26% to Median represented quartile 2 (Q2), Median to 75% represented quartile 3 (Q3) and 75% to Maximum represented quartile 4 (Q4).

**Table 4:** Association between quartile of BALF protein expression and clinical parameters.

Parameter	Quartile	MMP-1 (Std. dev.)	MMP-9 (Std. dev.)	MMP-12 (Std. dev.)	TIMP-1 (Std. dev.)
**Age**	1Q	67 (8)	62 (7)	61 (7)	63 (6)
	2Q	66 (7)	65 (8)	65 (8)	65 (8)
	3Q	67 (8)	**68** (7)	67 (3)	**68** (8)
	4Q	63 (7)	**68** (8)	**71** (5)	**68** (8)
**Pack years**	1Q	55 (22)	52 (26)	56 (24)	58 (29)
	2Q	57 (24)	55 (24)	56 (23)	59 (25)
	3Q	61 (37)	55 (21)	38 (6)	57 (37)
	4Q	53 (29)	64 (39)	57 (25)	53 (21)
**FEV1**	1Q	48 (12)	44 (11)	49 (13)	44 (13)
	2Q	47 (14)	48 (16)	46 (10)	47 (13)
	3Q	46 (13)	46 (13)	48 (15)	47 (12)
	4Q	45 (13)	49 (12)	52 (15)	48 (15)
**FEV1/FVC**	1Q	44 (10)	41 (8)	44 (10)	39 (9)
	2Q	41 (10)	43 (13)	42 (9)	44 (9)
	3Q	43 (9)	41 (9)	51 (7)	42 (10)
	4Q	41 (11)	45 (10)	45 (10)	43 (11)
**TLC**	1Q	112 (17)	115 (10)	117 (18)	117 (12)
	2Q	120 (14)	119 (18)	117 (15)	119 (17)
	3Q	116 (12)	113 (14)	109 (15)	115 (13)
	4Q	117 (17)	119 (16)	115 (14)	114 (18)
**CT Score**	1Q	36 (11)	40 (10)	41 (11)	41 (13)
	2Q	39 (12)	40 (16)	37 (11)	36 (12)
	3Q	35 (14)	37 (12)	21 (4)	35 (15)
	4Q	39 (15)	34 (12)	28 (14)	39 (11)
**St. George**	1Q	35 (18)	34 (14)	33 (13)	35 (10)
**Total**	2Q	40 (11)	38 (14)	41 (17)	39 (13)
	3Q	37 (13)	37 (13)	44 (11)	38 (16)
	4Q	37 (12)	41 (14)	39 (18)	37 (15)

The mean values of age, pack years, FEV1% predicted, FEV1/FVC % predicted, TLC % predicted, CT score and St. George questionnaire total score were calculated for each quartile of MMP-1, -9, -12 and TIMP-1 BALF protein expression. Bold indicates p<0.05 compared to quartile 1. Parentheses () indicate standard deviation.

**Table 5:** Association between quartile of plasma protein expression and clinical parameters.

Parameter	Quartile	MMP-1 (Std. dev.)	MMP-9 (Std. dev.)	TIMP-1 (Std. dev.)
**Age**	1Q	64 (8)	65 (7)	65 (8)
	2Q	67 (7)	68 (7)	66 (9)
	3Q	66 (8)	64 (7)	66 (6)
	4Q	66 (8)	66 (10)	66 (8)
**Pack years**	1Q	64 (27)	63 (29)	50 (22)
	2Q	50 (23)	52 (18)	47 (23)
	3Q	51 (22)	56 (24)	64 (38)
	4Q	62 (38)	56 (38)	64 (26)
**FEV1**	1Q	46 (12)	49 (15)	42 (10)
	2Q	45 (12)	46 (13)	49 (14)
	3Q	48 (14)	46 (14)	49 (13)
	4Q	48 (14)	47 (12)	48 (14)
**FEV1/FVC**	1Q	39 (8)	45 (10)	39 (8)
	2Q	42 (11)	42 (10)	43 (11)
	3Q	45 (10)	43 (11)	45 (9)
	4Q	42 (10)	40 (8)	42 (11)
**TLC**	1Q	116 (15)	115 (15)	118 (14)
	2Q	115 (15)	116 (13)	117 (14)
	3Q	117 (16)	111 (16)	113 (14)
	4Q	117 (15)	**124** (12)	116 (18)
**CT Score**	1Q	40 (13)	34 (14)	43 (11)
	2Q	39 (10)	37 (12)	38 (14)
	3Q	34 (12)	37 (13)	35 (11)
	4Q	37 (15)	41 (12)	35 (14)
**St. George**	1Q	38 (17)	37 (14)	36 (9)
**Total**	2Q	33 (11)	37 (13)	37 (12)
	3Q	38 (10)	35 (13)	35 (13)
	4Q	40 (15)	40 (14)	42 (18)

The mean values of age, pack years, FEV1% predicted, FEV1/FVC % predicted, TLC % predicted, CT score and St. George questionnaire total score were calculated for each quartile of MMP-1, -9 and TIMP-1 plasma protein expression. Bold indicates p<0.05 compared to quartile 1. Parentheses () indicate standard deviation.

**Table 6:** Coefficients of correlation (R^2^) between elastase and collagenase activity and COPD disease parameters.

	Elastase	Collagenase
Age	−0.03	−0.09
Pack years	0.13	0.00
FEV1	0.16	0.18
FEV1/FVC	0.05	0.00
TLC	0.18	0.12
CT score	0.01	0.11
D_L_CO	0.06	−0.26
St. George total	−0.13	−0.03

The coefficient of correlation (R^2^) was calculated for elastase and collagenase activity and age, pack years, FEV1, FEV1/FVC, TLC, CT score, D_L_CO and St. George total score.

PFT parameters are reported in % predicted post bronchodilator.

### Predictive Value of MMP Levels in COPD

**Figure 5:**
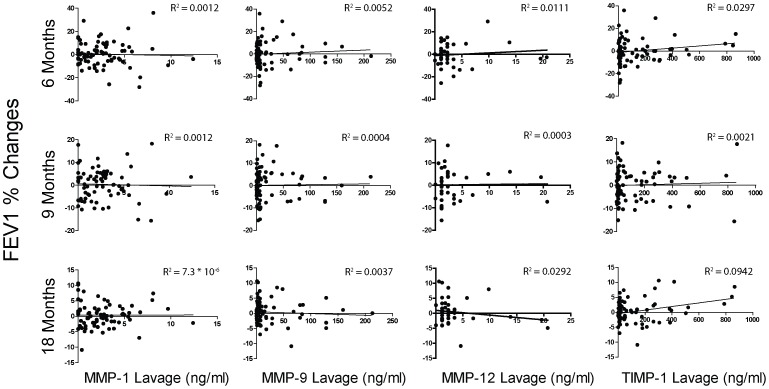
Correlation between BALF MMP/TIMP levels and % change in FEV1. BALF protein levels for MMP-1, -9 and TIMP-1 were correlated with the % change from baseline in FEV1 at 6, 9 and 18 months of follow up. The coefficient of determination (R^2^) was calculated for each value at each time point.

**Figure 6:**
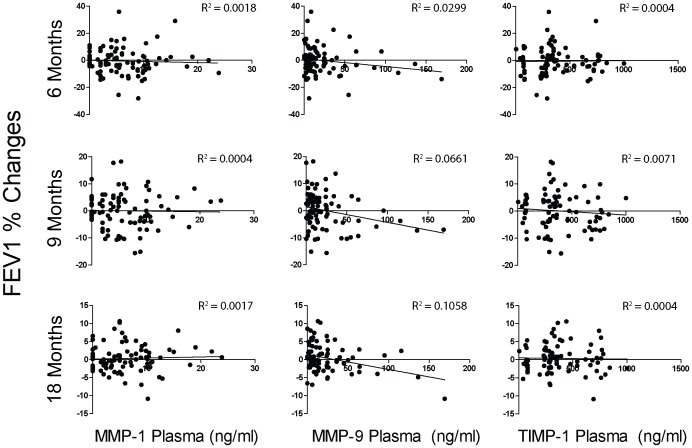
Correlation between plasma MMP/TIMP levels and % change in FEV1. Plasma protein levels for MMP-1, -9 and TIMP-1 were correlated with the % change from baseline in FEV1 at 6, 9 and 18 months of follow up. The coefficient of determination (R^2^) was calculated for each value at each time point.

To address whether MMP levels taken from a COPD subject were predictive of the future course of the disease, baseline MMP or TIMP values were correlated with the change in FEV1 over time. BALF (Figure 5) and plasma (Figure 6) baseline MMP and TIMP levels correlated poorly with the change in FEV1 at 6, 9 and 18 months follow up. Plasma MMP-12 was not analyzed since MMP-12 was not detectable in the plasma.

### Correlation between MMP BALF and Plasma Protein Levels in Emphysema

**Figure 7:**
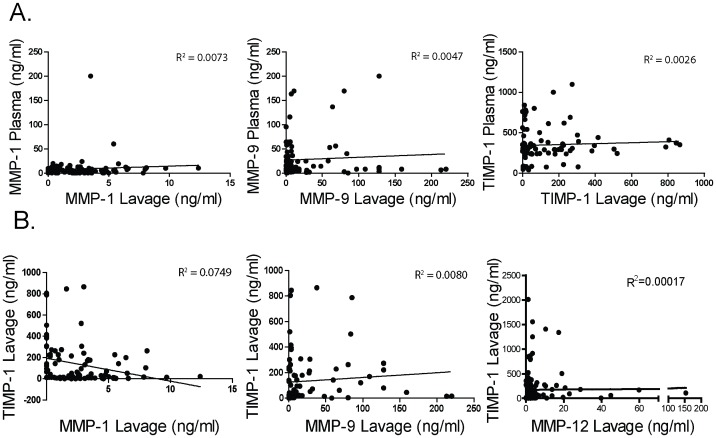
There is no correlation between BALF and plasma MMPs and TIMP-1 levels. ( A) Correlation between MMP/TIMP BALF and plasma levels. BALF protein levels for MMP-1, -9 and TIMP-1 were correlated with plasma MMP-1, -9 and TIMP-1 levels that were taken at the same time. The coefficient of determination (R^2^) was calculated for each value at each time point. (B) Correlation between MMP and TIMP-1 BALF levels. BALF MMP-1, -9 and -12 levels were correlated with BALF TIMP-1 levels that were taken at the same time. The coefficient of determination (R^2^) was calculated for each value at each time point.

Linear regression analyses compared simultaneous BALF and plasma MMP-1, MMP-9, and TIMP-1 measurements in COPD subjects (Figure 7A). BALF and plasma levels showed a poor correlation with each other. Linear regression analyses yielded an R^2^ coefficient of determination less than 0.01 for each association. Of note, these analyses were not conducted for MMP-12 since plasma levels of MMP-12 were undetectable.

### MMP/TIMP-1 Ratios in the BALF Samples

MMP/TIMP-1 ratios for MMP-1, -9 and -12 exhibited no significant difference between groups. Though the overall ratios of MMPs/TIMP-1 were not significantly altered, it is important to note that the correlation between BALF MMP and TIMP-1 levels was weak (Figure 7B). In fact, in the emphysema cohort, those with high MMP levels frequently had very low levels of TIMP-1 indicating that a proteolytic imbalance is likely present within the lung of the emphysema subjects.

## Discussion

The discovery of new markers that better correlate with disease severity and progression would provide important prognostic information for patients and physicians and greatly decrease the time and expense needed to demonstrate clinical benefit of a therapeutic agent [28]. Given the role of MMPs in the pathogenesis of this disease, we investigated whether MMP levels in the BALF or plasma could serve as a barometer of disease activity. This study demonstrates that MMP levels are increased in the BALF of patients with severe COPD; however, MMP levels by themselves did not correlate with disease severity and were not predictive of disease progression. Moreover, we found that baseline BALF and plasma levels correlated poorly with follow up measurements taken from the same individuals at a 3 or 6-month follow up period. Moreover, baseline MMP measurements did not correlate with changes in FEV1% predicted over an 18-month time period of follow up. Together, these results indicate that MMP levels in emphysema subjects are highly variable and have limited utility as a biomarker in this disease.

Though MMP levels were a poor prognostic predictor in this study, the increased MMP levels may reflect ongoing subtle disease activity not detectable with the standard available clinical measures over such a short period of time. Indeed, MMP levels may need to be tracked in a very large cohort for years to determine their effects on disease outcomes. Despite the high TIMP-1 levels, elastase and collagenase activity were significantly increased within the BALF of the emphysema cohort. What is interesting to note is that collagenase activity was only increased in the emphysema cohort. This was despite the fact that TIMP-1 levels were much higher in the emphysema cohort than the active smokers. This may mean that the dysfunctional matrix remodeling that leads to emphysema is not yet occurring in the smokers despite their increased MMP levels. It is well known that MMPs are increased in smokers even though not all these individuals will go on to develop COPD [29]. These findings suggest that the development of increased collagenolytic activity may represent a key transition to the disease state in chronic smokers. However, our results cannot address whether collagenase is a marker of smoke exposure intensity or disease susceptibility. This question will need to be explored in future longitudinal studies.

Unlike TIMP-1, MMP protein levels varied greatly in COPD subjects over time. This intra individual variability in MMP levels is unlikely to be due to technical factors. Bronchoscopy and plasma collection protocols were standardized across multiple centers and all samples were handled in a uniform manner. While variable return could affect lavage measurements, plasma MMP values, which are less subject to variations in collection, were also not stable in COPD subjects. Typically, baseline and follow up MMP measurements were batched together on the same plate and internal standards were used to ensure the reproducibility of our results. In fact, the ELISA analyses in these studies demonstrated a coefficient of variation of less than 20%. Importantly, emphysema subjects were in a steady state clinically and had no systemic steroid use and no history of recent exacerbation upon entry in the trial [23]. This shows that single point determinations may not reflect what is occurring in the lung over a prolonged period of time. Future biomarker studies will need to perform sequential testing in individual COPD subjects to address this important issue of temporal variability.

The decrease in plasma MMP-9 and TIMP-1 levels that were measured in the emphysema cohort contrasted with a recent study that showed that serum levels of these proteins were elevated in COPD [30]. It is important to note, however, that serum levels can be influenced by the release of MMPs following the degranulation of leukocytes and platelets during the ex vivo clotting process [31]. It is conceivable that circulating monocytes that produced these proteins may have been preferentially drawn to the lung thereby increasing lung while decreasing plasma levels.

Though this was one of the largest BALF and plasma analyses conducted in COPD, it is conceivable that our study size was not sufficient to detect the link between MMP levels and disease severity and progression. A limitation of the BALF studies was the large age discrepancy between the control subjects, smokers without emphysema and study group. Though we cannot rule out age as a confounding factor, MMP levels in control subjects were very low for MMP-1, -9 and -12 as has been reported in other studies of normal, non-smoking controls subjects [32], [33]. Thus, it is unlikely that these dramatic increases are due to age alone. Though measuring MMPs may not be useful for the majority of COPD patients, it may have a role for those with genetic polymorphisms that cause persistent increases in MMP expression [15], [34]. Future research will need to address whether those COPD subjects who do have stably high MMP levels undergo more progression of their disease. Indeed, polymorphisms in MMP-1, -9 and -12 have been reported to be associated with COPD-related phenotypes[14], [35]–[37].

In summary, these findings show that increases in BALF MMP levels are not associated with disease severity or progression. Moreover, these results demonstrate that MMP levels vary considerably over time even in stable COPD subjects. Importantly, this study showed that BALF collagenase activity was increased in emphysema and not in active “healthy” smokers. Further studies will need to examine whether the presence of increased collagenolytic activity within the lung heralds the development of disease in active smokers.
